# Flame monitoring and anomaly detection in steel reheating furnaces based on thermal video using a hybrid AI computer vision system

**DOI:** 10.1038/s41598-025-16276-y

**Published:** 2025-08-25

**Authors:** Jelle Vanhaeverbeke, Steven Verstockt, Sofie Van Hoecke

**Affiliations:** Ghent University-imec, IDLab, Ghent, 9052 Belgium

**Keywords:** Computer science, Information technology, Software

## Abstract

Reheating furnaces are essential in steel manufacturing, ensuring steel reaches the optimal temperature for hot-rolling. Burners within these furnaces produce flames to maintain the necessary thermal conditions. However, inconsistent burner performance can result in irregular or extreme flames, compromising steel quality and production safety. Traditionally, flame monitoring has relied on human supervision, which is inefficient and prone to errors. To overcome these limitations, we propose a computer vision-based system for automated flame monitoring and anomaly detection. The system analyzes the video stream from a thermal camera that continuously monitors the furnace interior. Our methodology involves three steps: (1) detecting flames and furnace keypoints using a deep learning model, (2) quantifying flames across burner regions with traditional computer vision techniques, and (3) identifying anomalies using an interpretable machine learning model. Validation with real-world data from a large steel manufacturing facility demonstrates that the system achieves an F1 score above 80% in detecting anomalies across various burner zones. To support operators, the results are presented in a dashboard that provides both real-time and historical insights into furnace performance. This enables timely anomaly detection and intervention, ensuring safe, efficient, and high-quality steel production.

## Introduction

The steel industry is a cornerstone of the manufacturing sector, producing essential materials such as steel sheets, bars, beams, and wires. These materials are important for a wide range of applications, from constructing buildings to manufacturing automobiles. The production of these steel base products, such as sheets and beams, involves a process known as rolling^1^, which shapes the steel into various forms with specific mechanical properties. Initially, steel is hot rolled by heating it above its recrystallization temperature and then passing it through a series of rollers to achieve the desired shape and dimensions. In some cases, the hot-rolled steel undergoes additional cold rolling to further refine its dimensions, surface quality, and strength.

The heating process prior to hot rolling is conducted in reheating furnaces equipped with multiple burners across various zones. Given the substantial heat generation involved, precise control is important, as excessive and unstable flame production can compromise steel quality and pose safety risks. Consequently, early detection of such events is essential to enable timely operator intervention. Traditionally, burner anomalies are identified through observing significant deviations in temperature measurements or conducting visual inspections of the furnace. However, this approach requires constant human supervision, which can be resource-intensive and may lead to missed anomalous events. To overcome these limitations, a thermal camera is installed inside the furnace which facilitates direct and automated monitoring of flames.

Several approaches can be employed to design such solution. Traditional computer vision algorithms can detect flames in the furnace, but practical implementation may encounter issues due to data variability, such as noise, illumination changes, and flame reflections on steel surfaces^2,3^. A more robust approach involves learning-based methods, like autoencoders, which detect anomalies from raw video footage^4,5^. Although they have shown to be effective, autoencoders offer limited insight into their decision-making process which is required for smoother adoption and acceptance by the operators. Alternatively, a semantic segmentation model can identify flames, allowing for anomaly detection based on the flame masks^6^, which has the benefit of providing visually explainable outputs.

In this work, we not only predict flame masks using a segmentation model but we introduce a three-step computer vision methodology for flame monitoring and anomaly detection. This approach integrates the segmentation model with traditional computer vision techniques to further refine flame predictions, assigning each flame to its respective burner region for more detailed spatial analysis. Additionally, we employ an interpretable machine learning model to detect anomalies within each burner region, providing operators actionable insights and increasing trust in the system’s decisions. By combining machine learning with traditional computer vision, this hybrid methodology leverages the strengths of both techniques.

The entire framework is integrated into a real-time monitoring and anomaly detection system designed for steel reheating furnaces. This system provides operators with a dashboard to monitor historical and real-time flame behavior across three zones, and receive alerts about anomalous flame production. As a result, operators can timely investigate and address potential issues, reducing the risk of damaged steel and improving production quality and consistency. The main contributions of this work include:

- a joint flame semantic segmentation and furnace keypoint detection model;
- a traditional computer vision pipeline to process flames and furnace keypoints, determining the flame quantity per burner region;
- decision stumps for fast and interpretable anomaly detection;
- a monitoring dashboard, indicating if, when, and where anomalies occur.

The following section provides an overview of related work on vision-based and sensor-based process monitoring within the steel industry. Next, Sect. “Data” introduces the dataset of thermal videos captured from the furnace, along with the corresponding ground-truth data used in this study. Building on this, Sect. “Methodology” details our proposed hybrid methodology for flame monitoring and anomaly detection. Afterwards, the results of both individual components and the integrated solution are presented and discussed in Sect. "Results and discussion". Finally, Sect. "Conclusions and future work" concludes the work and outlines potential directions for future research.

## Related work

### Vision-based monitoring in the steel industry

The steel industry involves numerous processes that require close monitoring to ensure product quality, production efficiency, and workplace safety. Among these, reheating furnaces play a key role in the processing of steel. Despite their importance, to the best of our knowledge, no prior research has proposed vision-based solutions specifically designed for monitoring the flame production in steel reheating furnaces. However, there has been considerable progress in developing vision-based systems for other types of furnaces used in steel manufacturing.

Zhang et al.^7^ introduced a vision-based system for analyzing flames in blast furnaces. Their work focuses on processing flame images to study temperature distributions and the flicker frequency of flames. The findings revealed significant temperature variations within raceways, which helped explain fluctuations in production quality and efficiency. This demonstrates the potential of flame analysis for improving furnace operations.

Similarly, Compais et al.^8^ developed a visual monitoring system for blast furnaces, but their work focused on estimating oxygen concentration in flue gases. Using images captured inside the furnace, they extracted features such as intensity and texture to train machine learning models, including logistic regression, support vector machines, and artificial neural networks. Their system enables early detection of abnormal combustion events, allowing operators to identify and address issues before they escalate, which aligns with the objective of our work.

In another study, Zhu et al.^9^ proposed a vision-based approach for monitoring the operational status of blast furnaces using burden surface video footage. Their system extracts and integrates multilevel features, handcrafted features from high-temperature gas images, and sequential video features into a monitoring network. The approach is effective in detecting abnormal conditions such as hanging, collapsing, and irregular gas flow, ensuring smooth and stable furnace operation.

Patra et al.^10^ developed a vision-based system for monitoring basic oxygen furnaces, specifically designed to detect slag in the tapping stream. Their approach leverages infrared imaging to distinguish slag from steel by using differences in emissivity between the two materials and generates alerts to minimize slag carry-over into ladles. Although their study focuses on slag detection rather than flame monitoring, their vision-based methodology also aims to ensure steel quality.

Further, Selim et al.^11^ concentrated on monitoring ladles in steel facilities. Their system uses thermal cameras to track ladle surface temperatures and identify ladle numbers. They combined traditional computer vision techniques with deep learning models such as Faster RCNN to provide a real-time monitoring solution capable of early anomaly detection. This combination of conventional and modern approaches is similar to our methodology, emphasizing practical and efficient monitoring systems.

### Sensor-based monitoring in the steel industry

In addition to visual methods for monitoring and anomaly detection, sensor data can also be used as input for processing algorithms and machine learning models. A closely related work by Thai et al.^12^ developed a flame monitoring system for steel reheating furnaces, focusing on optimizing burner performance. The researchers employed fiber-optic sensors to capture flame radiation characteristics over a wide range of wavelengths. They then used signal processing techniques in combination with a neural network to estimate two key burner performance indicators: excess air and nitrogen oxide emissions. Similarly, Bao et al.^13^ worked on optimizing reheating furnaces by developing a multivariate linear regression model aimed at predicting and regulating furnace temperature.

Beyond reheating furnaces, research has been conducted on monitoring blast furnaces. Zhou et al.^14^ employed principal component analysis (PCA) and independent component analysis (ICA) to monitor and diagnose abnormal furnace conditions. Meanwhile, Zhu et al.^15^ developed a fault monitoring algorithm that combines PCA with a Gaussian mixture model (GMM). Agrawal et al.^16^ contributed by estimating and visualizing the hearth liquid level in real-time, enabling operators to maintain better control and stability of the blast furnace.

### Conclusion

Many related studies have been conducted on vision-based and sensor-based analyses for monitoring various processes within the steel industry. While some sensor-based solutions for reheating furnaces share a similar research focus with our work, they primarily concentrate on optimizing the combustion process. In contrast, our research is dedicated to flame monitoring specifically for anomaly detection. To the best of our knowledge, there currently is no vision-based system that uses thermal video to monitor and detect anomalies of flames in steel reheating furnaces. This gap highlights the novelty and potential impact of our approach in enhancing safety and efficiency in steel production.

## Data

The required heat for hot-rolling steel is generated through a combustion process that produces flames. To monitor these flames, the partnering company installed an AXIS thermal camera positioned at the entrance of the furnace. Our access was limited to a continuous stream of video footage, which is stored in 5-minute segments as H.264 encoded videos for easier handling. Consequently, there is no accompanying metadata regarding the camera’s exposure settings or other configuration parameters.

### Data exploration

Examining the data reveals that a color palette has been applied to the thermal video footage, making it easier for humans to interpret. The palette used is similar to those employed by other cameras. Therefore, if a methodology can reliably process this common visualization of thermal images, it is adaptable to other thermal cameras as well.

**Fig. 1:**
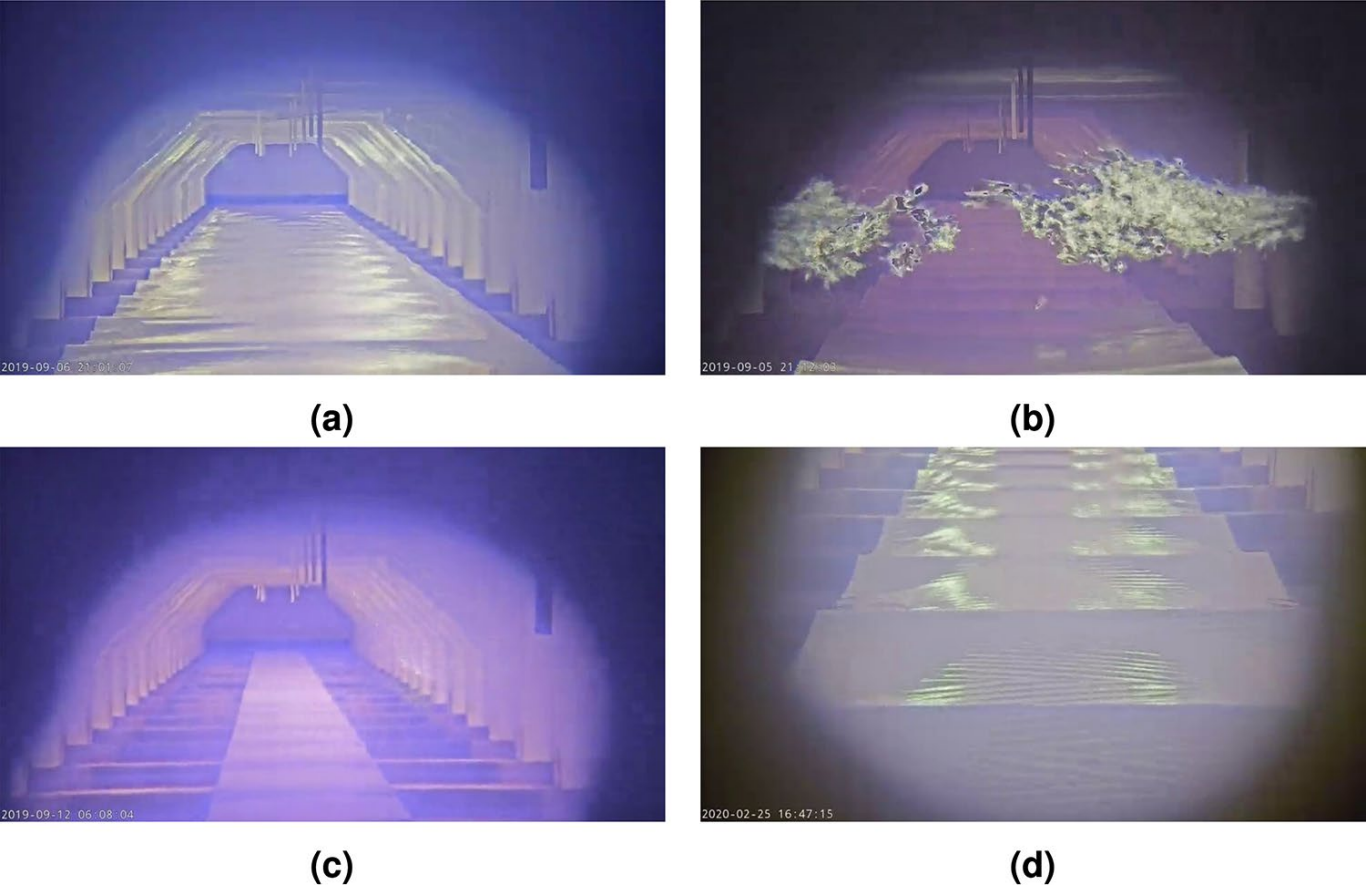
Example video frames illustrating the data variety, including (**a**) normal furnace operation, (**b**) high-intensity flames, (**c**) cold furnace, and (**d**) bad camera position.

Additionally, the collected data shows significant variation in flame, furnace, and camera characteristics, resulting in diverse visual appearances, as illustrated in Fig. 1. For example, it appears that the thermal camera automatically adjusts its exposure and/or tone mapping based on the intensity of the thermal radiation within the image to prevent oversaturation of high-intensity flames. While this adjustment preserves details in the flames, it simultaneously reduces the contrast in other areas of the furnace, as shown in Fig. 1b. Images can also exhibit low contrast in situations where the furnace is relatively cold, such as Fig. 1c.

Moreover, the camera’s position introduces further variability. The thermal camera is not entirely stable, exhibiting both short-term and long-term movement. Short-term variations include a constant, subtle up-and-down stuttering motion due to vibrations. Long-term movement involves changes in the camera angle over time, eventually requiring manual re-adjustment, as seen in Fig. 1d. Consequently, the camera’s position can also be changed by operator interventions.

These variations highlight the need for the designed methodology to be robust and capable of handling diverse conditions within the furnace environment.

### Labeled dataset

**Fig. 2:**
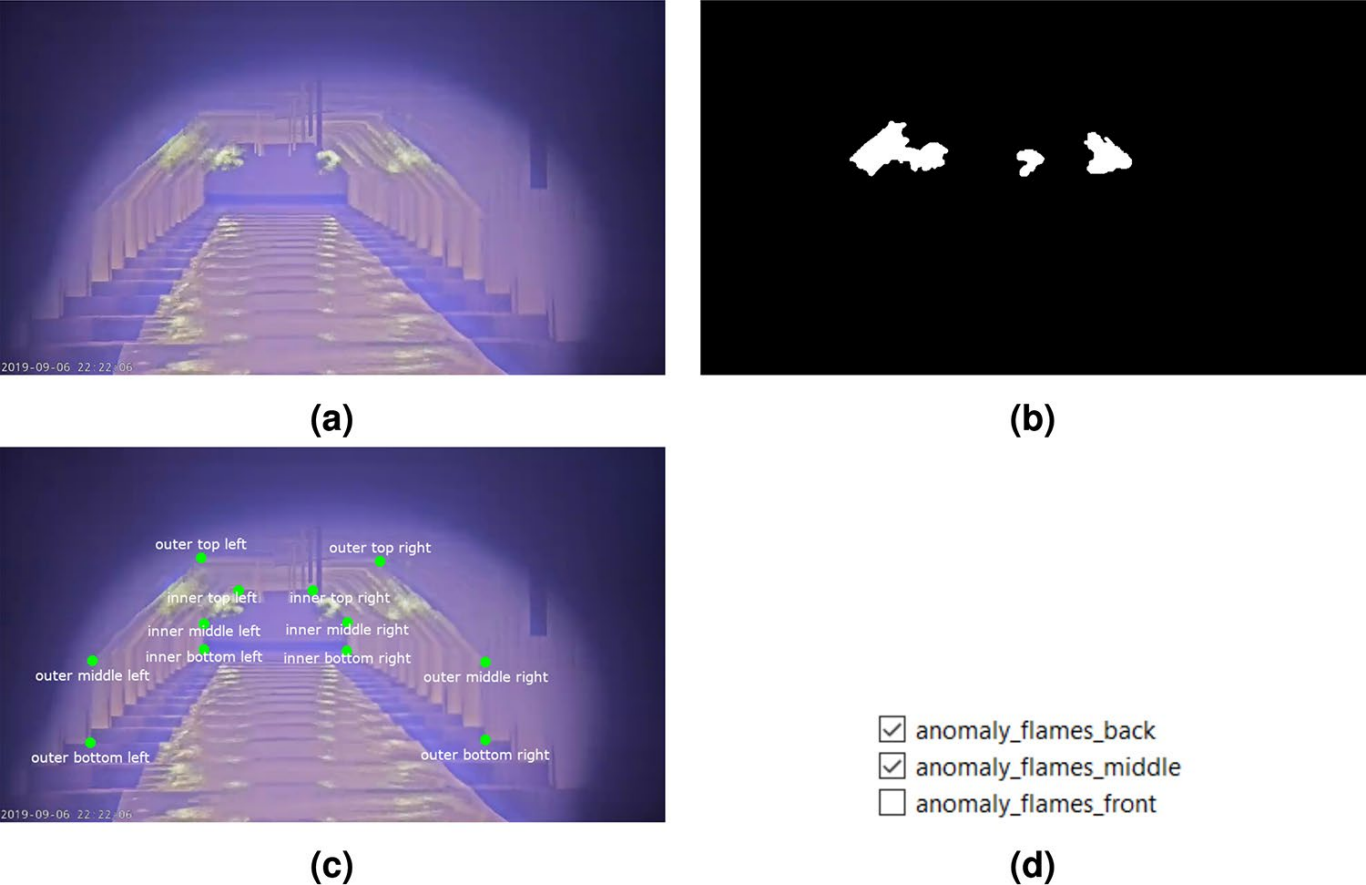
Example of the dataset illustrating (**a**) the thermal video frame, alongside (**b**) the corresponding flame mask, (**c**) furnace keypoints, and (**d**) flame anomaly status per burner region.

To create a dataset for training and validation of the various algorithms, a balanced and representative selection of videos is made. These videos show flames of different sizes under various furnace conditions. For each video, a subset of frames (e.g., Fig. 2a) is annotated with three types of labels per frame. First, a pixel-level flame mask is created (Fig. 2b). Second, 12 furnace keypoints are marked at important locations along the front and back edges of the furnace (Fig. 2c). Keypoints occluded by flames are excluded from the annotations. Since the camera position shifts over time, this keypoint labeling process is repeated for each frame. Finally, anomaly labels are added, indicating whether an anomaly is present in one of the three burner regions, i.e., front, middle, or back (Fig. 2d).

The labeling of the flames and keypoints is performed with high precision. To further ensure quality, all labels are reviewed by domain experts from the partnering company, who corrected inaccuracies when needed. Due to this rigorous labeling quality, the process required a significant effort per frame. Consequently, the final dataset consisted of 212 annotated frames sourced from 21 videos. Although the dataset size is relatively small, the frames encompass a wide range of conditions, making them highly representative of potential scenarios within the furnace. A robust methodology should be able to leverage this limited yet diverse dataset to its fullest potential.

The dataset is divided into a training and validation set, containing 137 and 75 frames, respectively. This split is carefully chosen to ensure both subsets are representative of the data’s diversity. To prevent bias in validation results, frames from the same video are not shared between the training and validation sets.

## Methodology

**Fig. 3:**
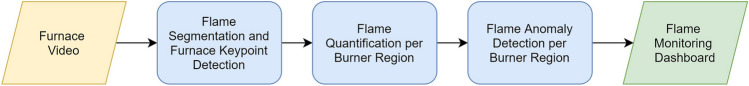
High-level overview of the furnace monitoring and anomaly detection pipeline.

The proposed anomaly detection methodology combines machine learning with traditional computer vision techniques, creating a hybrid approach that consists of three processing phases, as illustrated in Fig. 3.

In the first phase, a deep learning model is used to process thermal camera images, segmenting the flames within the furnace and detecting multiple furnace keypoints that serve as input for further analysis. In the second phase, the detected keypoints are processed using a traditional computer vision algorithm to construct three furnace regions, i.e., front, middle, and back. Subsequently, the segmented flames are assigned to their respective region and quantified, enabling more spatially fine-grained monitoring. In the final phase, anomaly detection is performed using a simple, fully interpretable machine learning model that evaluates the flame quantity in each region to identify anomalies. Dividing the methodology into multiple distinct phases ensures that the most suitable technique is applied to each step, resulting in an approach that is both accurate and capable of providing control and explainability for the generated results.

### Joint flame semantic segmentation and furnace keypoint detection

Using traditional computer vision algorithms to detect the flames is not feasible due to the challenging and varying data, including flame reflections, noise, and exposure changes. Hence, a deep learning model is designed and trained to perform two primary tasks: detecting all flames and identifying 12 furnace keypoints.

#### Model

**Fig. 4:**
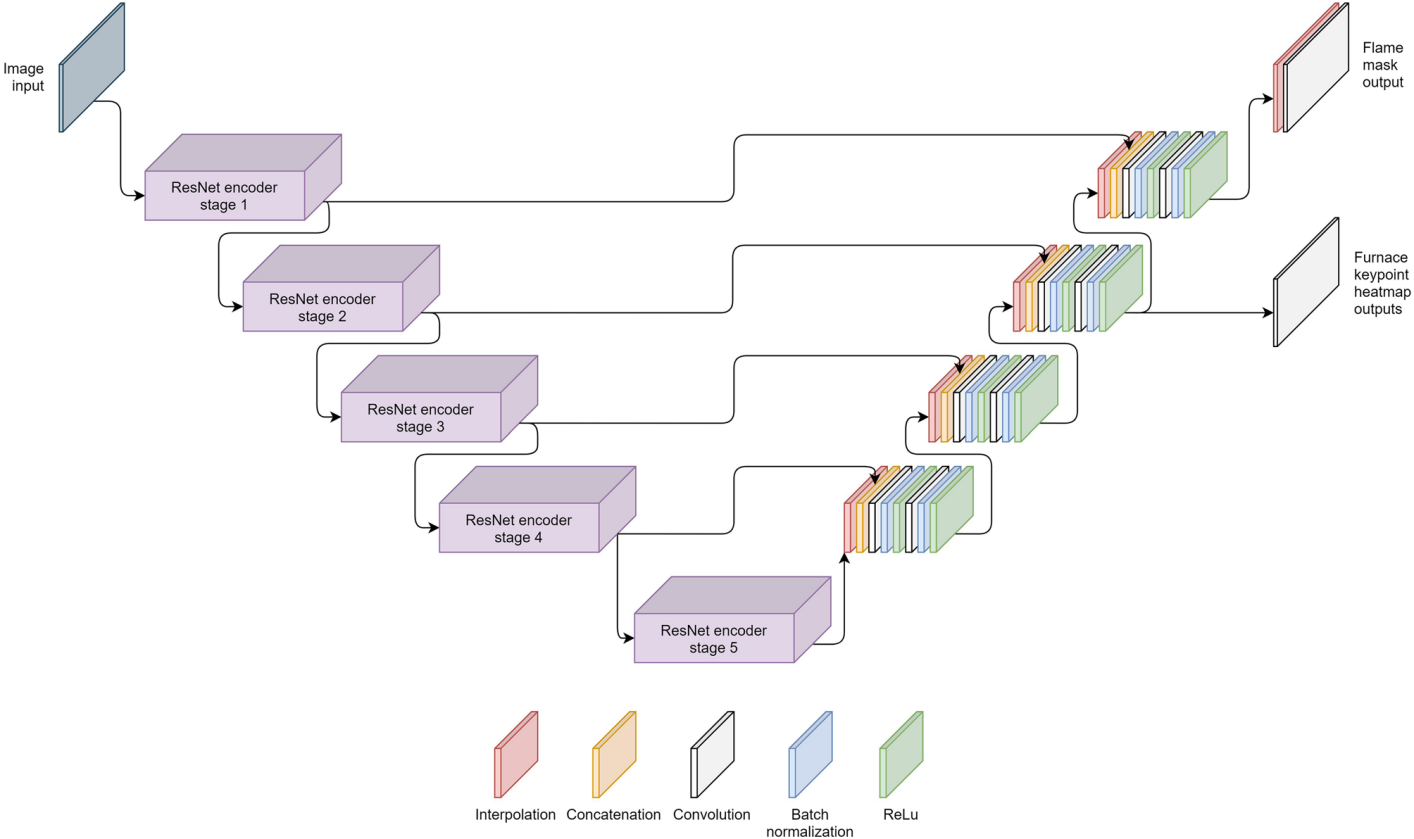
Architecture of the proposed joint semantic segmentation and keypoint detection model.

The model architecture consists of a pretrained ResNet^17^ encoder combined with a U-Net-style^18^ decoder featuring two output heads, as shown in Fig. 4. ResNet18, the smallest variant of the ResNet family, was selected as the backbone due to its balance of performance and efficiency. Larger configurations, such as ResNet34 and ResNet50, were also evaluated but showed comparable or slightly worse results. Additionally, ResNeSt50^19^, a ResNet-style network with improved layer structure, demonstrated marginally improved performance. Alternative backbone architectures, including VGG11^20^ and EfficientNetV2S^21^, were tested as well, yielding slightly better results than ResNet18. Despite these findings, ResNet18 was ultimately selected as the backbone due to its minimal performance differences compared to other architectures, which did not result in any significant qualitative impact. Additionally, ResNet18 provides faster inference speeds and a considerably lower parameter count, effectively reducing computational demands and reducing the risk of overfitting. It should be noted, however, that many of the other evaluated backbones demonstrated strong performance and could be viable alternatives depending on the specific requirements and priorities of the use case. For a full comparison of backbone results, refer to Appendix A.

The decoder used for the proposed model closely resembles the one used within the U-Net architecture, using skip connections from encoder to decoder in order to give it access to high-resolution information. The decoding blocks consist of an interpolation of the output of the previous block, concatenation with the output of the encoding block of the same resolution, a 3x3 convolution with batch normalization and ReLu, and a 1x1 convolution with batch normalization and ReLu.

To jointly predict the flame segmentation and furnace keypoints, the decoder is adapted to have two prediction heads, one for each task. The flame segmentation head is added at the top of the decoder and consists of an interpolation layer, to match the resolution of the input image, followed by a 1x1 convolution to predict the mask. The keypoint head does not regress the coordinates directly but predicts heatmaps containing a Gaussian peak since this is know to yield a higher accuracy and also gives extra insights in the prediction process of the model^22,23^. Therefore, the keypoint head does a 1x1 convolution outputting 12 channels, one for each keypoint location, based on the decoder features with a four times lower resolution. Afterwards, postprocessing of the heatmaps is needed to extract the coordinates of the keypoints.

#### Multitask loss function

The proposed model predicts both the flame segmentation and furnace keypoints in one forward pass. To learn this multitask prediction, a custom loss function is used that quantifies the error for both tasks.

The loss for the semantic segmentation task consists of two metrics, namely the cross-entropy and Jaccard loss. The cross-entropy loss optimizes for pixel-wise confidence, while the Jaccard loss aims for an optimal intersection of the predicted and target mask. We found that combining both losses made training more stable and better generalized for the use case at hand.

Training the model end-to-end on the error of the keypoint coordinates is not possible since they are predicted as heatmaps. Hence, the loss for the keypoint detection task is determined by calculating the mean squared error (MSE) between the predicted and target heatmaps. Using a regular MSE leads to slow and sub-optimal convergence since the size of the Gaussian peak is much smaller than the background area, resulting in a significant imbalance. This is solved by calculating a weighted MSE according to Equation (1)^24^.

1 $$\begin{aligned} WMSE = \frac{1}{n} \sum _{i=1}^n ((y_i - \hat{y}_i)^2 * (s * m_i + 1)) \end{aligned}$$

where:

$$\begin{aligned}\ WMSE&= \text {the weighted mean squared error (scalar)} \\ y \text { and } \hat{y}&= \text {the true and predicted values, respectively (tensor)} \\ m&= \text {the binary mask of keypoint regions (tensor)} \\ s&= \text {a scaling constant for the keypoint regions (scalar)} \\ n&= \text {the number of data points (scalar)} \end{aligned}$$

**Fig. 5:**
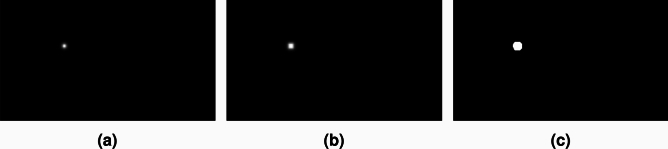
The weighting mask for the mean squared error is automatically generated by (a) obtaining the ground truth Gaussian peak, (b) applying a dilation, and (c) thresholding the result to create a binary mask of keypoint regions.

The weight mask is automatically generated by applying a small dilation on the target heatmap followed by a binary threshold, as visualized in Fig. 5. The weight multiplier is a hyperparameter telling how much attention must be given to the peak location. For this case, a value of 10 was found to give the best results. This weighted MSE loss does not ignore the background region completely but gives the Gaussian peaks a higher importance which helps the model to learn better and faster.

The losses for both tasks are then combined via a weighted sum, as shown in Equation (2). The cross-entropy and Jaccard loss are given an equal weight. However, since the keypoint MSE has a much lower range of values, it is assigned a higher weight of 20 to equalize the importance of both tasks. These weights are determined experimentally to optimize performance across both tasks.

2 $$\begin{aligned} ML = 0.5 * {CE}_{seg} + 0.5 * {J}_{seg} + 20 * {WMSE}_{kp} \end{aligned}$$

where:

$$\begin{aligned}\ ML&= \text {the multitask loss (scalar)} \\ CE_{seg}&= \text {the cross-entropy loss of the flame segmentation masks (scalar)} \\ J_{seg}&= \text {the Jaccard loss of the flame segmentation masks (scalar)} \\ WMSE_{kp}&= \text {the weighted MSE of the furnace keypoint heatmaps (scalar)} \end{aligned}$$

#### Data preprocessing and augmentations

**Fig. 6:**
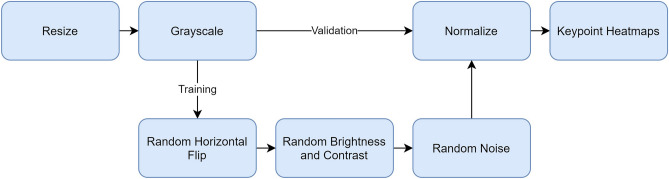
Data preprocessing and augmentation pipeline.

Several transformations and augmentations are applied to the data before feeding it to the model, as illustrated in Fig. 6. This process includes conventional transformations, such as resizing and normalizing the images, to ensure compatibility with an ImageNet-based pretrained backbone.

Additionally, the images are converted to grayscale. While the color palette applied to the original video frames enhances human interpretation, it introduces irrelevant and redundant information for deep learning models. This added complexity can cause the model to rely on color associations rather than focusing on the underlying patterns within the data. By converting the images to grayscale, this unnecessary complexity is removed, reducing the dimensionality of the input data and enabling the network to extract meaningful features more effectively. The benefits are evident in Appendix B, showing that applying grayscaling significantly improved validation results for both tasks, demonstrating that the model learned more relevant and robust features.

The keypoints require some preprocessing as well. Each keypoint is transformed into a heatmap featuring a Gaussian peak centered at the keypoint location, as depicted in Fig. 5a. For this study, 12 furnace keypoints are present, resulting in 12 heatmaps per image. Following previous research^25,26^, the Gaussian peak’s sigma is set to 2, providing a good balance between location accuracy and smoothness.

To increase data variation and regularization during training, runtime image augmentations^27^ are employed. These include random adjustments to brightness, contrast, and noise levels. Additionally, a custom random horizontal flip augmentation is used, which maintains the correct meaning of left and right. This is achieved by flipping the image along with its segmentation mask and keypoint coordinates while remapping keypoint labels according to their new positions. Although these augmentations do not further increase model performance, they play an important role in reducing overfitting and narrowing the generalization gap, as can be seen in Appendix B.

#### Training

The final model is trained for 100 epochs, which provides sufficient time for the model to converge. The training is executed in two stages. For the first stage, the ResNet18 backbone is kept frozen, and only the decoder is trained. After 75 epochs, the 3^rd^, 4^th^, and 5^th^ ResNet encoder blocks are unfrozen and fine-tuned which boosts the performance of the model a little more. Throughout the entire training process, the Adam optimizer is employed with a learning rate of 0.001. Other learning rates were tested but resulted in a slower or less stable convergence.

### Flame quantification per burner region

**Fig. 7:**
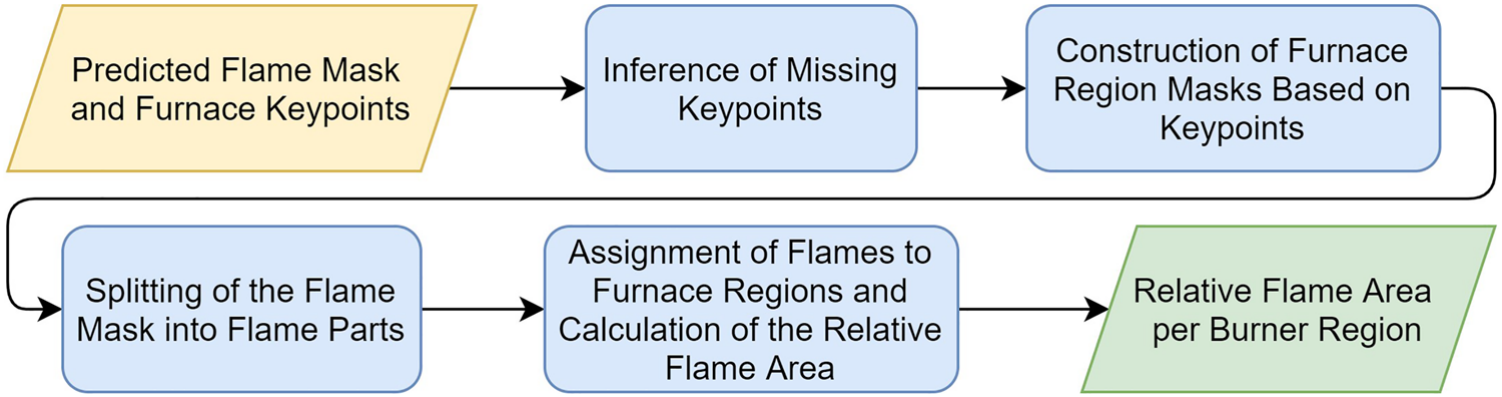
Overview of the computer vision pipeline for constructing the three burner regions and quantifying the flames per burner zone.

The previous section introduced a machine learning model designed to detect flames and furnace keypoints. In this section, the output of that model is further processed through a series of algorithmic and traditional computer vision steps, as summarized in Fig. 7. This processing pipeline is responsible for constructing the three furnace burner regions (i.e., front, middle, and back), assigning the flames to their respective regions, and quantifying the number of flames in each burner region. The details of each step in the pipeline will be explained in the following sections.

#### Inference of missing keypoints

Bad image contrast or occlusion by flames can lead to incorrect keypoint predictions. In order to recover some of those missing keypoints, a series of simple rule-based checks is applied relying on the rectangular relation of the *middle* and *bottom* keypoints. This relation allows us to make assumptions on the position of missing keypoints based on other known ones.

**Fig. 8:**
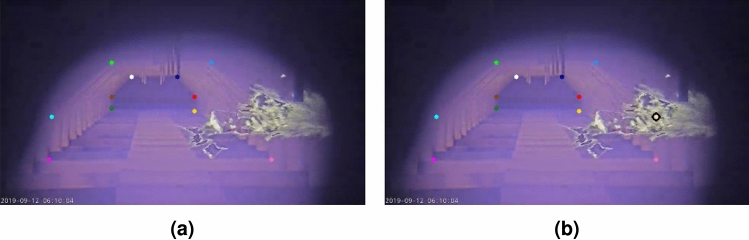
The *outer middle right* keypoint is (**a**) initially not predicted by the model but (**b**) successfully recovered using inference rules.

For example, when the *outer middle right* keypoint is missing and the *outer bottom right* and *outer middle left* keypoints are available, the *outer middle right* point can easily be inferred by combining the horizontal and vertical positions of the known keypoints, as shown in Fig. 8. However, this technique will not be able to recover keypoints if too many are missing.

#### Construction of furnace region masks based on keypoints

Ultimately, the flames in the front, middle and back region of the furnace must be quantified. Therefore, three region masks are generated based on the known furnace keypoints.

**Fig. 9:**
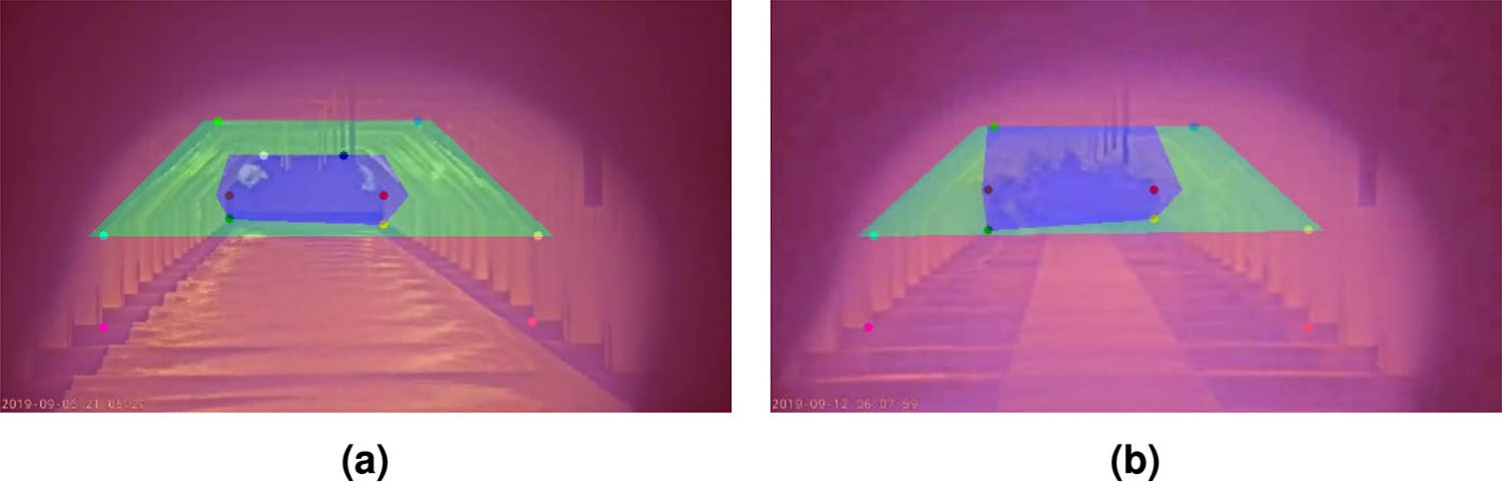
Examples of furnace region construction illustrating (**a**) the back (blue), middle (green), and front (red) burner regions when all keypoints are available, and (**b**) the fallback configuration for the back (blue) region in cases where the *inner top* keypoints are missing.

The back furnace region mask is primarily based on the information of the *inner* keypoints. If all of them are available, the region is built as the blue mask in Fig. 9a. The bottom of the region is exactly equal to the *inner bottom* keypoints, and the middle and top are slightly moved outward to better cover the flames in the back region.

The green mask in Fig. 9acovers the middle region of the furnace and is based on the four *top* and *middle outer* keypoints. The *outer bottom* keypoints are not included to build the mask since middle flames are not produced that close to the steel sheet. The complete region mask is however moved slightly outward to also cover flames that go just beyond the edge.

The front region mask (red in Fig. 9a) is the easiest to build. This mask covers the entire frame and will capture all flames that are not assigned to any of the other region masks.

**Fig. 10:**
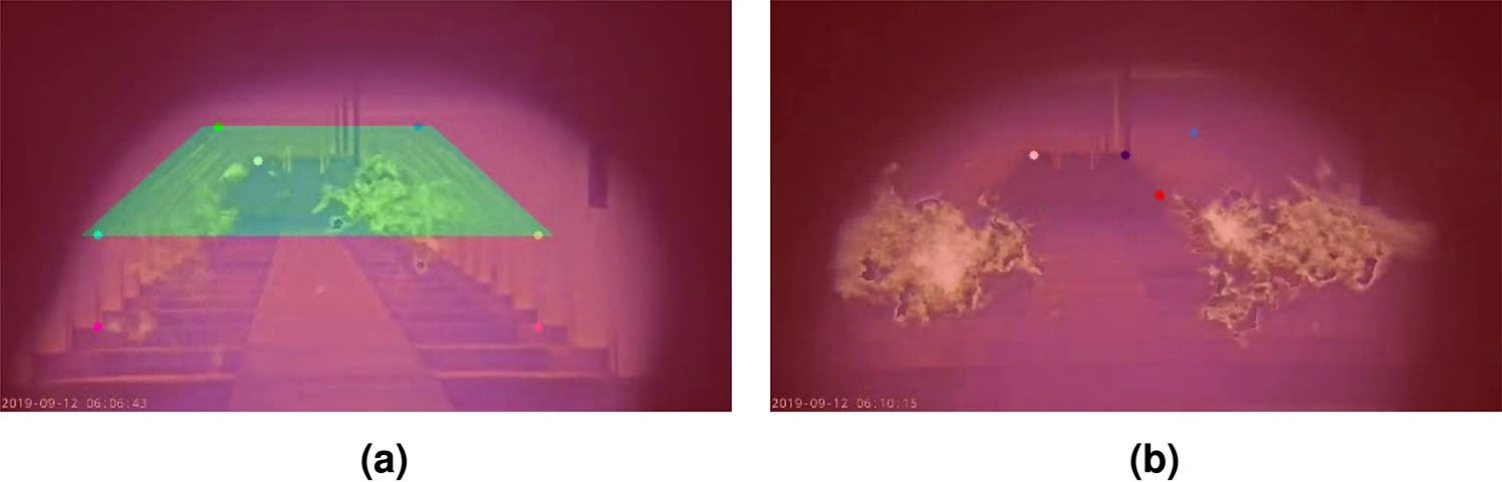
Examples of furnace region construction when too many keypoints are missing, resulting in (**a**) the absence of the back (blue) region and (**b**) the absence of both the back (blue) and middle (green) regions.

The procedure described above is executed when all keypoints are available, but this is not always the case. Even after the inference of missing keypoints, some might still be unavailable. To handle this, multiple fallback options are implemented for the different situations of missing keypoints that might occur. For example, when one of the *inner top* keypoints is missing, the back region is created based on the vertical position of the other known *inner top* keypoint and the horizontal positions of the *inner middle* and *bottom* keypoints. When both *inner top* keypoints are missing, the back region extends to the height of the *outer top* keypoints, as show in Fig. 9b. Similar fallbacks are implemented for the *outer top* keypoints. When too much keypoints are missing for the construction of a region mask, it is discarded completely. This is often not an issue since it only happens when large parts of a region are occluded with flames from regions in front of it. In Fig. 10a, the back region is not available because it is largely occluded by flames of the middle region, whereas in Fig. 10b, only the front region is available.

#### Splitting of the flame mask into flame parts

The model introduced in Sect. "Joint flame semantic segmentation and furnace keypoint detection" segments all flames in the image as one mask. As a consequence, the blobs of different flames can be merged if they are large or close together. Assigning those merged flames as a whole to one region would lead to incorrect results. Alternatively, assigning the flames pixel-by-pixel to their intersecting region would be inaccurate as well since some of the flames have offshoots into other regions. Therefore, this step in the pipeline aims to split the flame mask into multiple smaller flames that can be correctly assigned to their region afterwards.

**Fig. 11:**
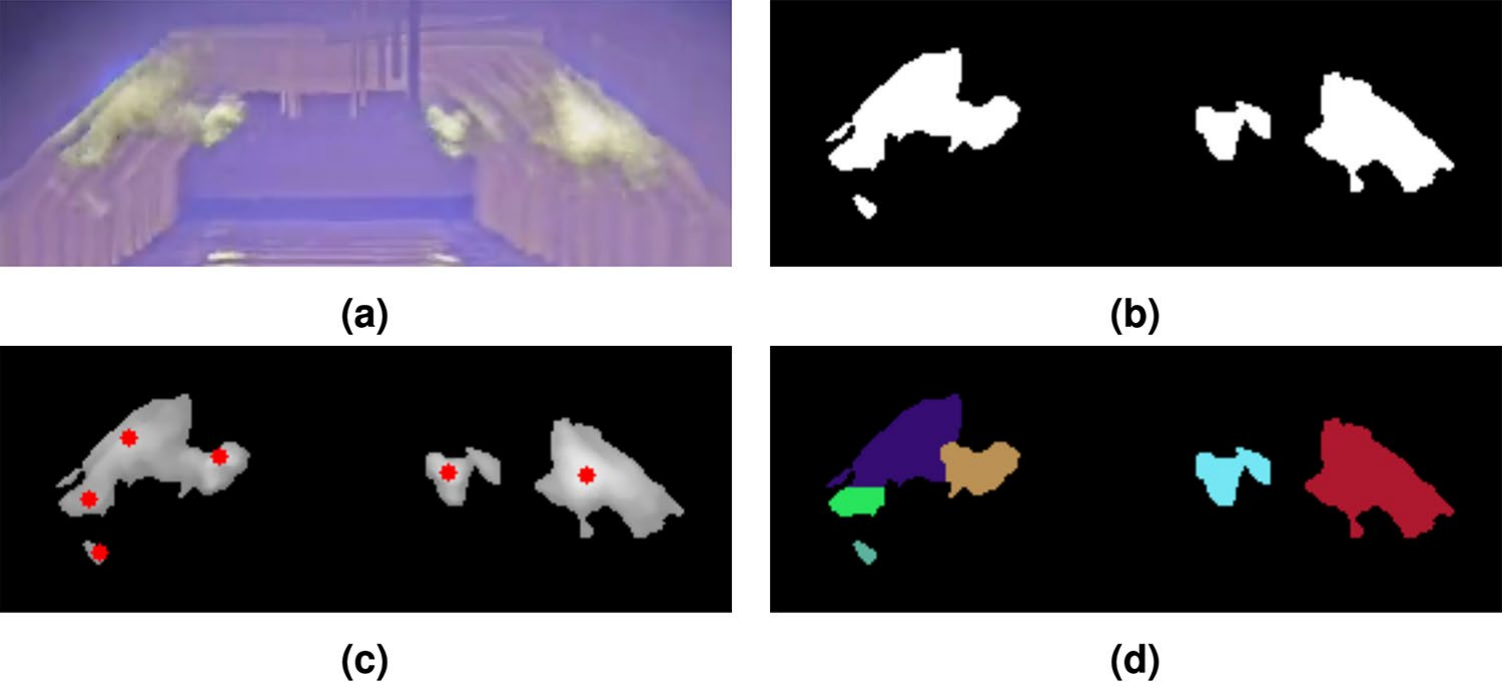
Visualizations of the flame splitting procedure, showing (**a**) the thermal image, (**b**) the flame mask, (**c**) the local peaks of the gray image, and (**d**) the flame mask split into regions using the Watershed algorithm.

Based on our inspection of the available thermal video footage, we observed that the core of the flame emits the highest levels of infrared radiation. This intensity gradually diminishes toward the flame’s edges, as illustrated in Fig. 11a. Similar findings have been reported in other research involving the imaging of flames in the infrared spectrum^28,29^.

This observation suggests that each high-intensity region corresponds to a different flame. Consequently, processing the intensity values by converting them to grayscale and applying a light median blur produces an ideal input for the Watershed transform^30^. This algorithm works by interpreting intensity values as a topographic landscape that is progressively “flooded” until the boundaries of different basins meet. These boundaries then define the segmentation regions. The seeds required for the Watershed transform are also determined from the grayscale image by identifying the local peaks.

An example of this procedure can be seen in Fig. 11. The mask of two flames on the left are undesirably merged into one chunk (Fig. 11b). After finding the local peaks (Fig. 11c) and performing the Watershed transform as described above, the flame is spit into multiple flame parts, separating the overlapping flames (Fig. 11d). Sometimes, too many splits are made, such as the bright green part in the example. Yet, this is not an issue since they will be assigned to the proper region in the next stage of the pipeline.

#### Assignment of flames to furnace regions and calculation of the relative flame area

**Fig. 12:**
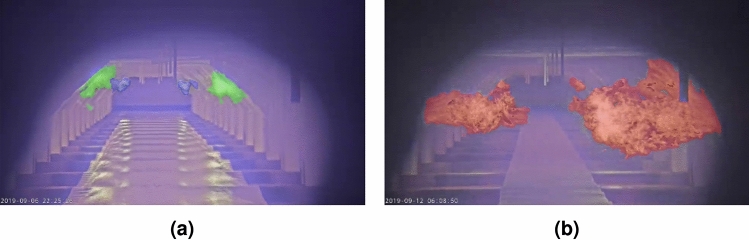
Examples of flame assignments, showing (**a**) flames allocated to the middle and back regions and (**b**) flames allocated to the front region.

Once the furnace regions are constructed and the flame masks are split, the flames can be assigned to their corresponding furnace region. This assignment begins by calculating the intersection area between each flame and the various burner regions. A flame is then allocated to the region with which it shares the largest intersection. This procedure is repeated until all flames have been assigned to a specific region, as illustrated in Fig. 12a.

To easily monitor the behavior of flames over time, the relative flame area for each burner region is calculated. This is done by dividing the flame area by the area of its respective burner region. Using relative rather than absolute flame areas provides a more robust measurement that is less sensitive to variations in camera placement.

Additionally, an extra rule is implemented to improve the accuracy. If the relative flame area of the front burners exceeds a certain threshold, occlusion may prevent accurate assignment of flames to the middle and back regions. In such cases, all flames are reallocated to the front region, as shown in Fig. 12b. The flame areas are then summed, and the relative flame area is recalculated. Although this adjustment may decrease the precision of the algorithm, it enhances its overall stability in practical applications.

### Flame anomaly detection per burner region

The methodology outlined in the previous section quantified the flames in each burner region. Building on this, the final phase of the proposed methodology leverages this information to perform automatic anomaly detection using a lightweight and interpretable machine learning model. This automated anomaly detection plays a crucial role in alerting operators to any irregularities without requiring them to continuously monitor the furnace. These alerts enable operators to promptly investigate and address potential issues, ensuring smooth furnace operation and preventing damage to the steel.

#### Model

**Fig. 13:**
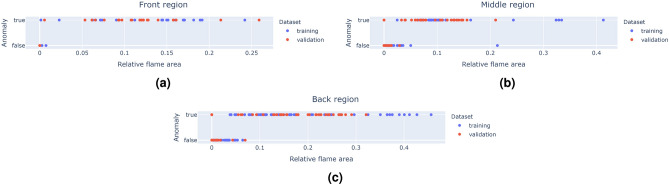
Scatter plots depicting the relationship between relative flame area and anomaly labels across the entire dataset for (**a**) the front region, (**b**) the middle region, and (**c**) the back region.

Figure 13 illustrates the relative flame area of frames across the complete dataset, plotted against their anomaly ground truth for the three burner regions. These plots demonstrate a clear distinction in flame area between normal and anomalous samples, indicating that the relative flame area is a highly informative feature for detecting anomalies. Notably, some samples labeled as anomalous have a relative flame area of zero. In such cases, the corresponding burner region could not be defined based on the available keypoints, leading the flames to be assigned to the region in front and contributing to the anomalous state over there.

Given the high discriminatory power of the relative flame area, a simple model suffices for anomaly classification. For this task, decision stumps are selected, which are a type of decision tree^31,32^ characterized by a single split or decision node. Decision stumps classify data into two groups based on a single feature and threshold, making them lightweight, fast, and, most importantly, fully interpretable.

#### Training

As described in Sect. “Data”, the thermal camera frames in the dataset are labeled with three anomaly states, one for each burner region. Each state is binary, indicating whether an anomaly is present in that region or not. These labels serve as the basis for constructing the decision stumps.

**Fig. 14:**
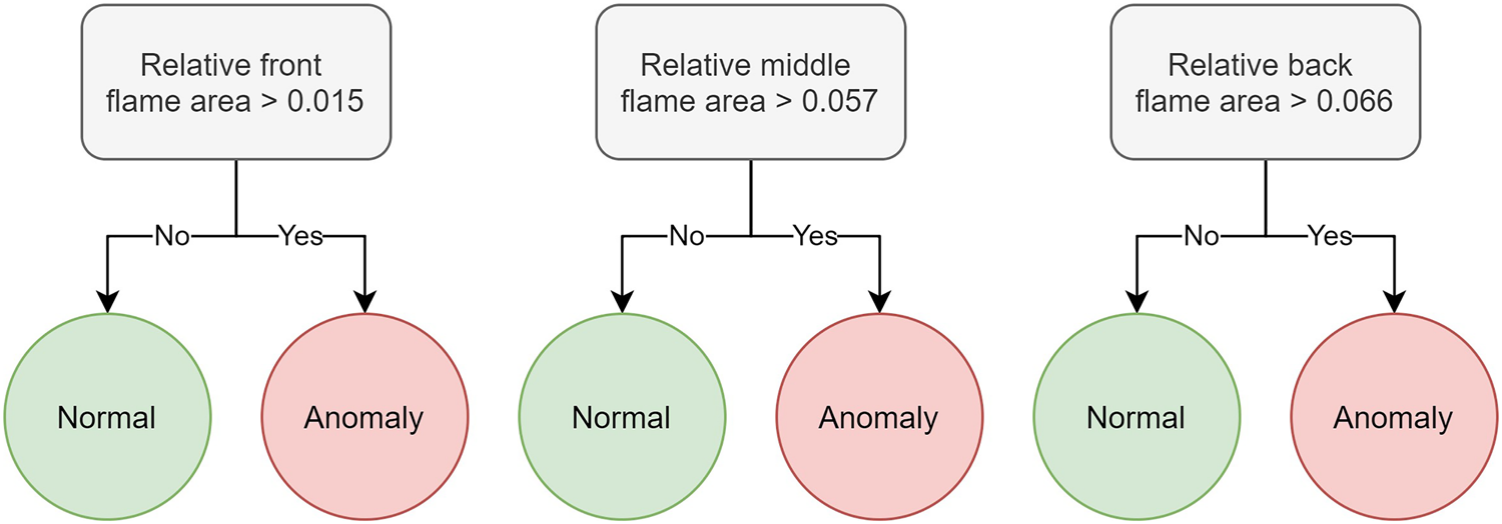
Graphical visualization of the three trained decision stumps, one for each burner region (front, middle and back).

A separate decision stump is created for each region, as a single stump would fail to generalize effectively across all three zones, as can be seen in Fig. 13. The resulting decision stumps, designed to predict the anomaly status per region, are shown in Fig. 14.

## Results and discussion

This section presents and discusses the results in three parts. First, the performance of the flame segmentation and furnace keypoint detection model is evaluated and analyzed. Second, the anomaly detection results are presented, providing insights into the performance of the complete methodology. Finally, the integration of the methodology into a dashboard is described, including an overview of its visualizations and processing speed on standard hardware.

### Joint flame semantic segmentation and furnace keypoint detection

Table 1 presents the performance of the final flame segmentation and furnace keypoint detection model evaluated using two metrics. The Jaccard index, which is the complement of the Jaccard loss used during training, validates the flame segmentation task. For keypoint detection, the percentage of correct keypoints (PCK) metric is used, as it offers a more intuitive validation compared to mean squared error (MSE). The PCK metric assesses whether the predicted keypoints lie within a specified distance from the target keypoints. In this study, this distance threshold is set to 1% of the image width. For both metrics, higher scores indicate better performance.

**Table 1:** Training and validation metrics of the final joint flame segmentation and furnace keypoint detection model.

**Jaccard index** **(flame segmentation)**		**Percentage of correct keypoints** **(furnace keypoint detection)**	
**Training**	**Validation**	**Training**	**Validation**
87.6%	82.8%	94.3%	90.7%

The model demonstrates solid performance with validation results exceeding 80%. The generalization gap between training and validation scores is limited, suggesting that the model is well-fitted and robust despite being trained on a small dataset. This is attributed to the use of a low-parameter model and relevant augmentations. To illustrate how these metrics translate into practice, several real-world examples are discussed below.

**Fig. 15:**
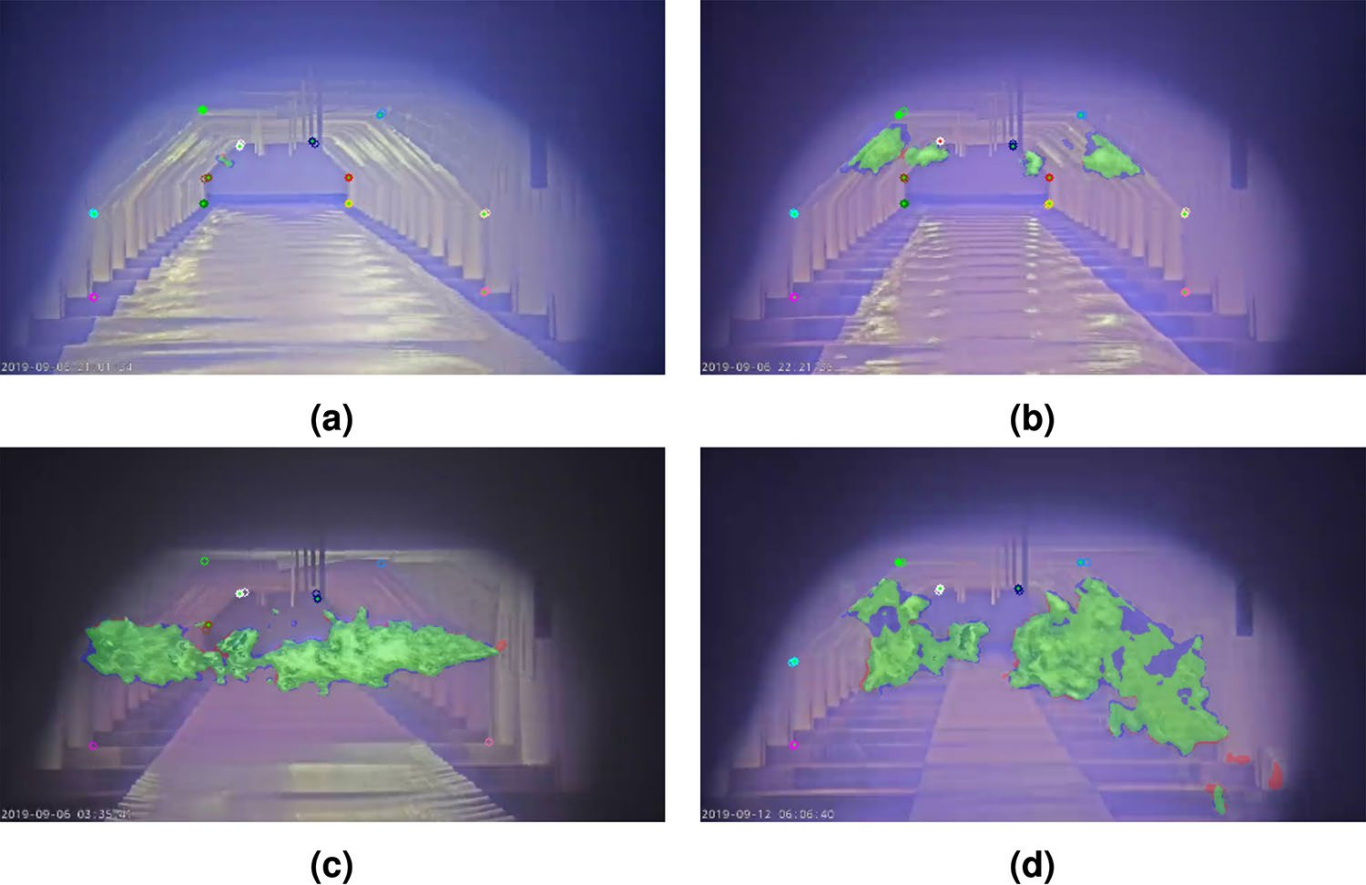
Example model predictions on varying furnace conditions. The segmentation mask is visualized using three colors: green represents a true positive (TP) prediction, blue represents a false negative (FN) and red is a false positive (FP). The correctness of the 12 furnace keypoints is visualized using two circles each. The outlined circle shows the target location whereas the solid circle visualizes the predicted keypoint. The small red or green dot within this circle tells whether the prediction is seen as correct or not according to the PCK metric.

In Fig. 15a, an example of normal furnace operation is presented, characterized by controlled and limited burner flames. The model accurately predicts the flame mask, even in cases where the flames in the back are small, and successfully identifies all keypoints. Figure 15b depicts a scenario where the burners are operating more intensively, which may suggest potential instability in furnace operation. Despite this, the model effectively predicts both the flame mask and keypoints. However, one keypoint, the *inner top left*, is considered incorrect by the PCK metric since it was not annotated due to occlusion caused by the flames. Nonetheless, the prediction demonstrates the model’s strong generalization capabilities.

Figure 15c showcases a situation where the front burners are active, which is undesirable as it could damage the steel. While the flame segmentation remains accurate, the limited visibility and reduced contrast result in missing keypoints. In the final example, shown in Fig. 15d, there is excessive flame production across all burners. In this case, the flame segmentation accuracy is slightly lower because parts of the flames are highly transparent. Additionally, many keypoints are occluded, although the visible ones are correctly predicted.

In summary, the model performs almost perfectly under normal furnace operating conditions. However, excessive flame production can reduce visibility and contrast in thermal images, leading to less accurate keypoint predictions. Although augmentations such as brightness and contrast adjustments improve the model’s robustness, they cannot fully overcome the challenges, as even human interpretation would be difficult under these conditions. Nevertheless, even when predictions are imperfect, such as in Fig. 15c and d, subsequent pipeline steps can extract meaningful insights, as the presence of an anomalous event is evident. Missing keypoints are not necessarily problematic since their absence also conveys useful information, indicating that flames in preceding regions are causing occlusion, and it is more accurate to avoid constructing regions behind these flames altogether. Finally, an important observation is that reflections on the steel sheets are not mistakenly classified as flames, further demonstrating the reliability of the model.

### Flame anomaly detection per burner region

Building on the results of the flame segmentation and furnace keypoint detection model, this section assesses the anomaly detection performance of the proposed methodology. Table 2 provides a comparison of the training and validation F1 scores for the decision stumps applied to the three burner regions. These scores are calculated using two different strategies. The first approach leverages the ground truth flame mask and furnace keypoints, offering a more focused assessment of the anomaly detection performance. In contrast, the second approach employs predicted flame masks and furnace keypoints to evaluate the end-to-end performance of the proposed system.

**Table 2:** Anomaly detection performance per burner region evaluated in two ways. First, using the ground truth flame mask and furnace keypoints giving a more isolated view on the performance of the anomaly detection. Second, using the trained model to predict the flame mask and furnace keypoints giving an end-to-end view on the performance of the proposed system.

**Burner region**	**F1 score**			
	**Using ground truth flame** **mask and furnace keypoints**		**Using predicted flame** **mask and furnace keypoints**	
	**Training**	**Validation**	**Training**	**Validation**
Front	97.4%	97.7%	97.4%	97.7%
Middle	90.0%	87.9%	93.5%	80.6%
Back	90.9%	88.8%	92.7%	83.1%

When using the ground truth data, the front region flames are classified with high accuracy, achieving a validation F1 score of 98%. However, the middle and back regions exhibit slightly lower performances, with validation F1 scores of 88% and 89%, respectively. Overall, this shows a robust classification capability of the anomaly detection model based on the relative flame area.

In the end-to-end evaluation scenario, where predicted data is used, the front region maintains its high validation F1 score of 98%. However, there is a noticeable decline in performance for the middle and back regions, with scores dropping to 81% and 83%, respectively. This decrease can be attributed to the dependence on keypoints that are not consistently available when predicted by the model. The front region does not experience this drop in performance because its classification does not rely on keypoints for construction of its region.

In all cases, the anomaly detection F1 score for the front region consistently surpasses those of the middle and back regions. This can be explained by the larger size of front flames, which creates a more pronounced distinction between normal and anomalous operations, thereby simplifying the classification task. Despite some challenges in the middle and back regions, these results show the effectiveness of using decision stumps to detect flame anomalies.

### Flame monitoring dashboard

**Fig. 16:**
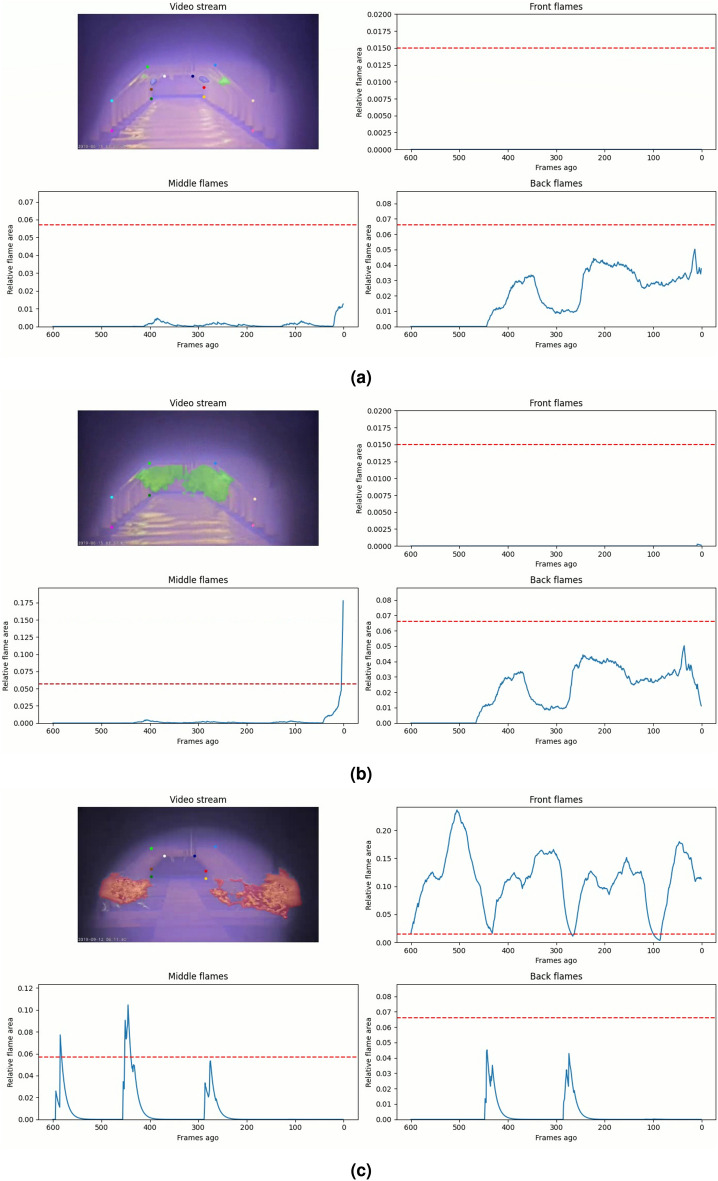
Examples of the monitoring dashboard during different furnace conditions, including (**a**) normal furnace operation, (**b**) sudden overactivity of middle burners, and (**c**) uncontrolled front flames.

The presented methodology is integrated as a system, going from video footage to anomaly detection, which visualizes the results through a dashboard for operators. 2 frames per second are processed to reduce the computational needs and allow for real-time execution on CPU. This is however sufficient to produce an insightful data stream to monitor the furnace. Some examples of the furnace monitoring dashboard can be seen in Fig. 16. The interface is divided into four sections visualizing different pieces of information. The top left corner shows the current processed frame of the video stream together with the predicted furnace keypoints and flames assigned to their respective region. The other three plots present the relative flame area over time for the front, middle and back burner regions. The data of these plots, in blue, are smoothed exponentially to make the signal more stable while the horizontal red line denotes the anomaly threshold learned by the decision stumps of Sect. "Flame anomaly detection per burner region". This allows the operator to easily see if, when and how much the flame area exceeds the threshold.

A normally operating blast furnace usually has some small and controlled flames in the middle and back to keep the furnace at temperature, as seen in Fig. 16a. This is also reflected by the time series plots of the middle and back relative flame area which have rather low values, far below the anomaly threshold. Sudden blasts of flames and burners that work too hard are clearly noticeable in the data, as is the case for the middle flames in Fig. 16b. Large flames in the front of the furnace are never desired. Figure 16c shows such an event where the data clearly shows the intervals and amount of flames that were detected in the front of the furnace.

## Conclusions and future work

The production of flames in steel reheating furnaces often deviates from the expected combustion process, which can impact efficiency and quality. To address this issue, a computer vision system has been developed to monitor flames and detect anomalies in three distinct burner zones using thermal camera footage. This system comprises three main components. First, a joint semantic segmentation and keypoint detection model is employed to extract a flame mask and identify 12 keypoints within the furnace. Second, a traditional computer vision pipeline quantifies the flames in each burner region. Third, decision stumps are utilized to detect anomalies in flame production. These components are integrated and displayed on a monitoring dashboard for real-time analysis.

The system’s performance has been validated with promising results, demonstrating its potential application in an industrial setting. The flame segmentation model achieved a Jaccard index of 82.8%, while the furnace keypoints were predicted 90.7% correct (PCK). The end-to-end anomaly detection evaluation yielded F1 scores of 97.7%, 80.6%, and 83.1% for the front, middle, and back regions, respectively. Despite these strong results, the model occasionally struggles with low-contrast thermal images. However, this challenge does not significantly affect the overall effectiveness of the monitoring and anomaly detection outputs.

Several possibilities for future research exist. Currently, only one furnace is equipped with a thermal camera. While every aspect of the proposed methodology was designed to be as generic and robust as possible, expanding the dataset to include footage from additional furnaces would allow for validation across different environments. Additionally, exploring deep learning architectures that exploit the temporal nature of the video stream could further enhance model performance.

In conclusion, this work enables the automatic monitoring of steel reheating furnaces. The promising results demonstrate the system’s potential in the steel industry, contributing to more efficient manufacturing and consistent product quality.

## Supplementary Information

Supplementary Information.

## Data Availability

The data used in this study are not publicly available due to commercial sensitivity. However, access to the dataset may be granted by the partner company upon reasonable request and may be subject to a data-sharing agreement. Additionally, a sample of the dataset is available at https://github.com/predict-idlab/flame-monitoring-anomaly-detection.

## Code availability

The code supporting the findings of this study is not publicly available due to commercial sensitivity. However, it can be obtained from the corresponding author upon reasonable request.
