# Supplementary material for: Flame monitoring and anomaly detection in steel reheating furnaces based on thermal video using a hybrid AI computer vision system
Source: Sci Rep. 2025 Aug 25;15:31300. doi: 10.1038/s41598-025-16276-y (PMC12379151; doi:10.1038/s41598-025-16276-y)
Supplement: Supplementary file 1 — Supplementary Information. [file 41598_2025_16276_MOESM1_ESM.pdf]

Appendix A Flame Semantic Segmentation and Furnace Keypoint Detection Results using Different Backbones

	Jaccard index (flame segmentation)		Percentage of correct keypoints (furnace keypoint detection)		Inference speed	Number of parameters
**Backbone**	**Training**	**Validation**	**Training**	**Validation**	**Intel Core i7-8650U**	
ResNet18	87.6%	82.8%	94.3%	90.7%	**3.2 FPS**	**14M**
ResNet34	87.5%	83.4%	93.8%	89.1%	2.5 FPS	24M
ResNet50	87.2%	81.1%	93.7%	90.3%	0.7 FPS	73M
ResNeSt50	84.0%	**84.3%**	93.6%	91.1%	0.4 FPS	75M
VGG11	88.4%	83.6%	95.7%	**92.0%**	1.5 FPS	19M
EfficientNetV2S	88.9%	83.2%	96.5%	91.1%	1.5 FPS	32M

Appendix B Flame Semantic Segmentation and Furnace Keypoint Detection Results using ResNet18 Backbone with Different Preprocessing and Augmentations

	Jaccard index (flame segmentation)		Percentage of correct keypoints (furnace keypoint detection)	
**Preprocessing**	**Training**	**Validation**	**Training**	**Validation**
Resize and normalize	91.1%	79.3%	97.5%	81.7%
+ Grayscale	91.5%	**83.1%**	97.3%	89.1%
+ Random horizontal flip, brightness, contrast and Gaussian noise	87.6%	82.8%	94.3%	**90.7%**
